# Support for relatives in the intensive care unit: lessons from a cross-sectional multicentre cohort study during the COVID-19 pandemic

**DOI:** 10.1186/s12913-023-09756-2

**Published:** 2023-07-18

**Authors:** Sophie C. Renckens, H. Roeline Pasman, Hanna T. Klop, Chantal du Perron, Lia van Zuylen, Monique A. H. Steegers, Birkitt L. ten Tusscher, Floor C. H. Abbink, Wouter de Ruijter, Lilian C. M. Vloet, Stephanie C. E. Koster, Bregje D. Onwuteaka-Philipsen

**Affiliations:** 1 Department of Public and Occupational Health, Amsterdam UMC, Location VU Medical Center, Amsterdam, The Netherlands; 2 Expertise Center for Palliative Care Amsterdam UMC, Amsterdam, The Netherlands; 3 Viaa University of Applied Sciences, Zwolle, The Netherlands; 4 Department of Medical Oncology, Cancer Center Amsterdam, Amsterdam UMC, Location VU Medical Center, Amsterdam, The Netherlands; 5 Department of Anaesthesiology, Amsterdam UMC, Location VU Medical Center, Amsterdam, The Netherlands; 6 Department of Intensive Care Medicine, Amsterdam UMC, Location VU Medical Center, Amsterdam, The Netherlands; 7 Department of Paediatrics, Amsterdam UMC, University of Amsterdam, Amsterdam, The Netherlands; 8 Department of Intensive Care Medicine, Noordwest Ziekenhuisgroep, Alkmaar, The Netherlands; 9 Foundation Family and Patient Centered Intensive Care (FCIC), Alkmaar, The Netherlands; 10 Research Department of Emergency and Critical Care, HAN University of Applied Sciences, Nijmegen, The Netherlands; 11 Department of Anaesthesiology and Department of Intensive Care Medicine, Zaans Medisch Centrum, Zaandam, The Netherlands

**Keywords:** Family Centred Care, COVID-19, Relatives, Critical care, Family support, ICU

## Abstract

**Background:**

Support for relatives is highly important in the intensive care unit (ICU). During the first COVID-19 wave  support for relatives had to be changed considerably. The alternative support could have decreased the quality and sense of support. We aimed to evaluate how support for relatives in Dutch ICUs was organised during the first COVID-19 wave and how this was experienced by these relatives in comparison to relatives from pre-COVID-19 and the second wave. Additionally, we aimed to investigate which elements of support are associated with positive experiences.

**Methods:**

We performed a cross-sectional multicentre cohort study in six Dutch ICUs in the Netherlands. Written questionnaires were distributed among relatives of ICU patients from pre-COVID-19, the first wave and the second wave. The questionnaire included questions on demographics, the organisation of support, and the experiences and satisfaction of relatives with the support.

**Results:**

A total of 329 relatives completed the questionnaire (52% partner, 72% woman and 63% ICU stay of 11 days or longer). Support for relatives of ICU patients during the first COVID-19 wave differed significantly from pre-COVID-19 and the second wave. Differences were found in all categories of elements of support: who, when, how and what. Overall, relatives from the three time periods were very positive about the support. The only difference in satisfaction between the three time periods, was the higher proportion of relatives indicating that healthcare professionals had enough time for them during the first wave. Elements of support which were associated with many positive experiences and satisfaction were: fixed timeslot, receiving information (e.g. leaflets) on ≥ 2 topics, discussing > 5 topics with healthcare professionals, and being offered emotional support.

**Conclusions:**

Although, support for relatives in the ICU changed considerably during the COVID-19 pandemic, relatives were still positive about this support. The altered support gave insight into avenues for improvement for future comparable situations as well as for normal daily ICU practice: e.g. daily contact at a fixed timeslot, offering video calling between patients and relatives, and offering emotional support. ICUs should consider which elements need improvement in their practice.

**Supplementary Information:**

The online version contains supplementary material available at 10.1186/s12913-023-09756-2.

## Background

Support for relatives and communication are important pillars of Family Centred Care [1], which is gaining increasing importance in the intensive care unit (ICU) [2]. In the ICU, communication between relatives and ICU healthcare professionals is one of the most highly valued aspects of perceived quality of care by relatives and central in shaping relatives’ experiences throughout the ICU admission [3–5]. Unsatisfactory communication from healthcare professionals in the ICU towards relatives, such as a lack of quality of information, empathy, support, and use of non-verbal cues has been found to be associated with an increased risk of post-ICU burden [4, 6]. Furthermore, relatives’ satisfaction with ICU experience is known to be influenced by the availability and quality of emotional and social support by healthcare professionals [7, 8].

The ways in which support was provided to relatives of ICU patients was forcedly changed during the first COVID-19 wave. Due to a combination of the high volume of patients being cared for, severe shortage of personal protective equipment and the visitation restrictions that were in place [9, 10], ICUs shifted from in-person meetings with relatives towards relying almost exclusively on tele-communication, most often via telephone and sometimes using video calling. This could potentially have decreased the quality of communication and sense of support [11]. There were different ways in which ICUs organised this alternative support. In many ICUs, the ICU healthcare professionals continued to provide support to relatives via tele-communication, while other ICUs established dedicated teams for the support for relatives [12, 13]. These so-called family support teams (FSTs) often consisted of non-ICU medical specialists from different departments and specialties [12–14]. These alternative ways of support mostly ended after the first COVID-19 wave.

There is ample evidence from before the COVID-19 pandemic about important elements of support for relatives of ICU patients, such as structured communication, usage of information brochures, and multidisciplinary support [15, 16]. A number of studies has been published on how support for relatives of ICU patients was organised during the COVID-19 pandemic in the United Kingdom and Scandinavia [12, 17], as well as qualitative studies on relatives’ experiences with the support they received in France, the Netherlands, the United States and Canada [11, 13, 18, 19]. However, to the best of our knowledge, no quantitative studies have disentangled the altered ways of support into basic elements of support for those relatives and investigated how these elements relate to the experiences of relatives. Therefore, this study aimed to evaluate how the support for relatives in Dutch ICUs was organised during the first COVID-19 wave and how this was experienced by these relatives in comparison to relatives from before COVID-19 and the second COVID-19 wave. During the second wave most visitation restrictions were lifted and alternative ways of support ended. In addition, we aimed to investigate which elements of support are associated with positive experiences with the support, which helps us to formulate recommendations for the future comparable situations, as well as for normal daily practice in the ICU.

## Methods

### Design and study population

We performed a cross-sectional multicentre cohort study using written questionnaires in six Dutch ICUs in the North-Western part of the Netherlands. Two of the six ICUs were located in two affiliated academic hospitals, and the other four were general hospitals. Three ICUs used newly developed FSTs, whereas in the other three ICUs the ICU healthcare professionals continued providing the support, yet via tele-communication. First contact persons of ICU patients from three different time periods (pre-COVID-19, first COVID-19 wave and second COVID-19 wave) were eligible if they fulfilled all of the inclusion criteria and none of the exclusion criteria. There was one first contact person per patient. Detailed in- and exclusion criteria are shown in Table 1.

**Table 1:** In- and exclusion criteria

Inclusion criteria First contact person of an intensive care unit (ICU) patient with the following inclusion criteria:			
	**Pre-COVID-19**	**First COVID-19 wave**	**Second COVID-19 wave**
**Age patient**	≥ 18 years	≥ 18 years	≥ 18 years
**Period of ICU stay**	December 1, 2019 – February 1, 2020	March 15 – May 15 2020	October 1, 2020 – January 1, 2021
**Length of ICU stay**	≥ 3 days	≥ 3 days	≥ 3 days
**Diagnosis**	N/A	Confirmed COVID-19 infection	Confirmed COVID-19 infection
**Other criteria**	Invasive mechanical ventilation ≥ 3 days	N/A	N/A
Exclusion criteria			
First contact person has insufficient proficiency of the Dutch language			

*N/A* Not applicable

### Description of family support teams

Three ICUs within our sample used one or more Family Support Teams (FSTs) during the first COVID-19 wave from mid-March until mid-May 2020. Most of the key elements of the FSTs were similar among the three ICUs. The FSTs consisted of physicians who were not part of the clinical ICU team and were from a variety of medical specialties: e.g. oncology, anaesthesiology and geriatrics. In one ICU, the FSTs also assisted in turning COVID-19 patients from supine to prone position and vice versa. All FSTs worked under authority of the treating physician. The FSTs provided the daily support to relatives via telephone giving primarily clinical updates about the patient and sometimes discussing the well-being of the relative. Critical decisions, such as stopping respiratory support were communicated by the treating physician. FSTs were informed about the situation of the patient via the electronic medical records and in some cases it was possible that they attended multidisciplinary consultation meetings. In one ICU, FST members also attended the daily ward round. One ICU had a psychosocial support team in addition to the FST. This team consisted of spiritual caregivers, medical social workers and psychologists, to whom the FST members could refer if relatives needed additional psychosocial support.

### Data collection

Medical records of ICUs were automatically searched for eligible patients by local ICU contact persons using a standard query created by CdP, following an additional manual eligibility check by CdP and SCR. If the patient was eligible, the contact information of the first contact person was abstracted, as well as some patient’s characteristics. Relatives who met the inclusion criteria were approached by telephone between January and July 2021 for participation in the questionnaire study. This was on average 10.5 months (range 4–18 months) after the patients’ ICU admission date, which was longest for the pre-COVID-19 group (mean 15 months) and shorter for the first and second COVID-19 wave (respectively mean 11 and 6 months). A maximum of three attempts to reach a relative by telephone were made, in which study information was provided and a short eligibility check was performed. If relatives provided consent to receive the study information and written questionnaire per mail, this was sent to them within 7 days. Relatives were asked to consent to study participation at the start of the written questionnaire. Relatives were sent reminder letters after three and six weeks if they had not yet responded.

### Measurement

The current study is part of a larger study, which means that other topics were also covered in the questionnaire such as treatment decision-making, well-being of relatives and support for relatives in the period around the end-of-life of a patient (Additional file 1). Between 74 and 103 questions were included in the questionnaire, depending on the personal situation of the relative (e.g. patient deceased or not), and the expected time investment was 40–60 min. The questionnaire was pilot tested among representatives of a Dutch ICU patient and relative organisation, and revised based on their input. A number of questions from the questionnaire were included in the analyses for the current study and will be discussed below.

#### Relative and patient demographic characteristics

Medical record abstraction was performed by CdP and SCR for two patient’s characteristics: gender and whether the patient died in the ICU. In the written questionnaire, relatives were asked about additional patient’s characteristics, including age, ICU length of stay, and whether the patient had been transferred to or from another ICU. Additionally, questions on relative’s demographic characteristics were asked, including kinship to the patient, gender, age, level of education, cultural background and whether they had a COVID-19 infection during the patient’s ICU admission.

#### Elements of support and visitation policy

The questionnaire included a number of questions on how the support was organised, which can be categorised into four groups. The first category is *who* provided the support, which included the type of healthcare professional that relatives received support from. This category was supplemented with a variable on whether relatives from the first COVID-19 wave received support from a FST, which was known based on information of an ICU coordinator at each study site. The second category contains data on *when* relatives received support, including items on the frequency of contact and whether relatives had a fixed timeslot at which they received support. The third category is on *how* the support was provided and included a question on the method of contact between relatives and healthcare professionals. The last category is about *what* kind of support relatives received, including items on the topics that relatives received information on (e.g. leaflets), the topics that were discussed in conversations with healthcare professionals, the possibility of video calling with the patient and whether relatives were offered emotional support. For the analyses of associations between elements and experiences, the number of topics was included, instead of individual topics that relatives received information on or were discussed. Finally, relatives were asked if they were allowed to visit the patient in the ICU and relatives of deceased patients were asked if they were allowed to say goodbye in person and if this could be done in a private room.

#### Experience and satisfaction with support

For the current study, 11 items of the written questionnaire related to experiences and satisfaction with support were used. Five items were derived from the validated Consumer Quality Index Relatives in the ICU (CQI R-ICU), which measures the perceived quality of care from the perspective of patients’ relatives [5]. The items covered whether the relative received comprehensible information from healthcare professionals, whether they received contradictory information from healthcare professionals, whether they felt taken serious by healthcare professionals, whether healthcare professionals had enough time for them, and whether healthcare professionals listened carefully. All items could be scored as never, occasionally, usually, always or not applicable, which was dichotomised for analysis purposes into never/occasionally and usually/always, while not applicable was treated as a missing value. In addition, six self-developed items were included. Relatives were asked whether they were satisfied with the frequency of the support they received (yes/ no, preferably more often/ no, preferably less often) as well as with the timing of the support they received (yes/ a little/ no). The answer options were dichotomised into yes (respectively yes, and yes and a little) and no (remaining answer options). Relatives were also asked to rate attending ICU nurses, attending ICU physicians, non-ICU healthcare professionals and psychosocial caregivers for the support they gave on a scale of 1 to 10. Finally, relatives of deceased patients were asked to rate the support they received during the last phase of life of the patient.

### Statistical analysis

IBM SPSS Statistics 26 was used for statistical analyses. Descriptive statistics were used to describe the sample characteristics, the elements of support and visitation policy, and relative’s experiences and satisfaction. This is summarised for the total population and for relatives from the three time periods separately. Continuous data were summarised using medians (interquartile ranges (IQR)) whereas categorical data were summarised using frequencies (percentages). Differences between relatives from the three time periods were tested using chi-squared tests for categorical data and Kruskal–Wallis H tests for continuous data. Chi-squared tests were replaced by Fisher’s exact tests when > 20% of the cells of a contingency table had an expected count of less than five. Likewise, differences were tested between relatives from the first COVID-19 wave who primarily received support from a FST and relatives from the first COVID-19 wave who received support from ICU healthcare professionals, and between relatives with different periods between ICU admission and study participation (5–6 months, 7–12 months, > 12 months). All tests were two-tailed and an alpha level of 0.05 was used. Associations between thirteen elements of support (independent variables) and seven experience and satisfaction outcomes (dependent variables) were analysed pairwise using logistic regression analyses, which were adjusted for age and gender of the relative. Considering the multiple analyses on the same dependent variable in the logistic regression analyses, we applied a Bonferroni correction to minimize the chance of Type 1 error. The resulted in a Bonferroni adjusted alpha level of 0.0038 for the logistic regression analyses.

### Ethics

Relatives were informed about the study both orally and in writing. All relatives provided written informed consent before filling in the questionnaire. The questionnaire included a note on the potential emotional burden of re-calling recent experiences with an ICU admission, including the contact details of an independent healthcare professionals who was available for consultation. The Medical Ethics Review Committee of VU University Medical Center determined exception from formal review under Dutch law (registration number 2020.0618). Additionally, institutional review boards at each site approved all procedures (Dijklander Science Centre and Board of Directors Dijklander Ziekenhuis (DOC 020), Board of Directors Ziekenhuis Amstelland (n.s.), Board of Directors Zaans Medisch Centrum (HF21038), Science Office Noordwest Ziekenhuisgroep (L021-037)). The study complied with the Netherlands Code of Conduct for Scientific Practice from the Association of Universities in the Netherlands (VSNU).

## Results

**Fig. 1:**
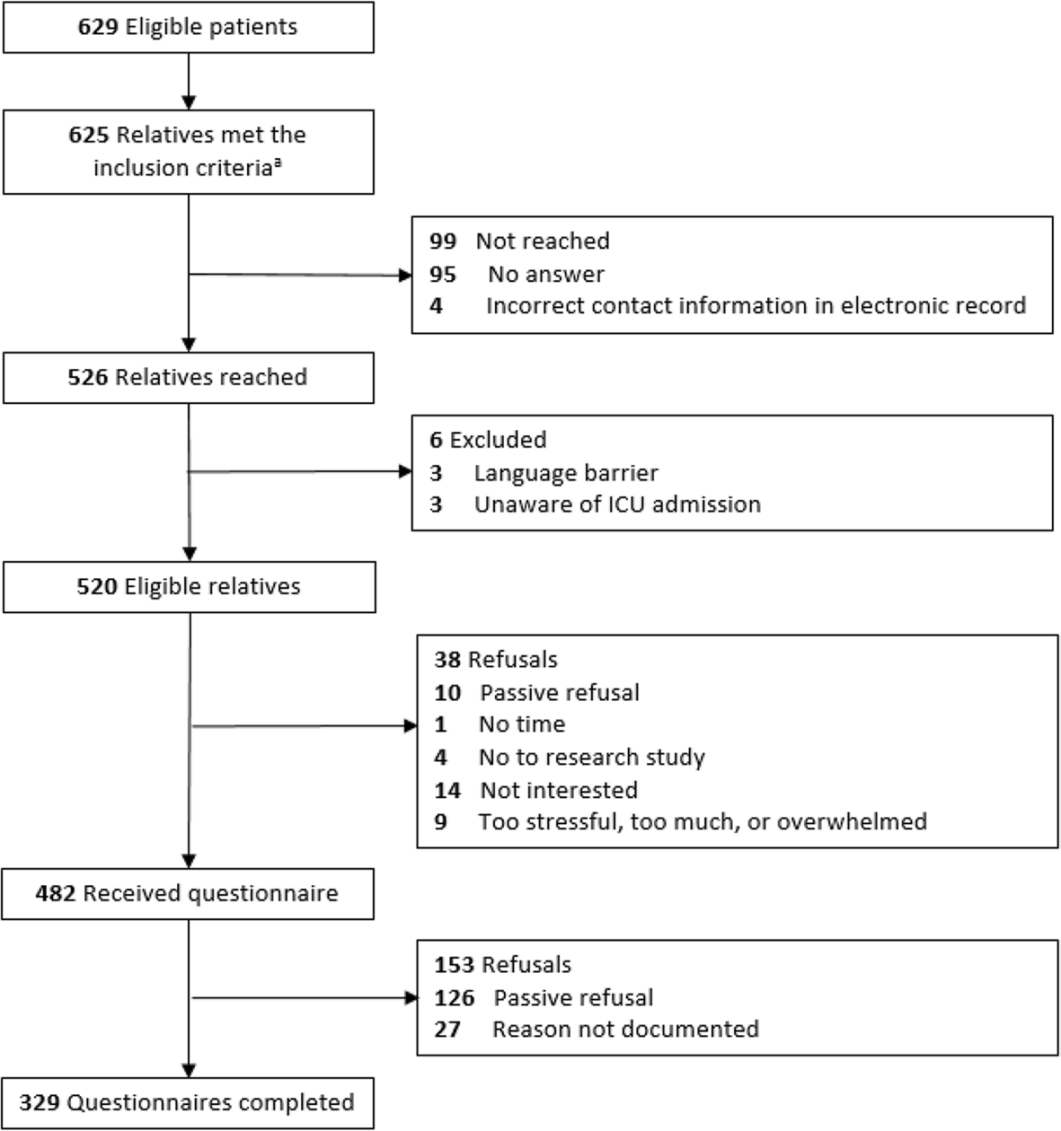
Eligibility and enrolment flowchart

A total of 625 relatives fulfilled the inclusion criteria, of whom 526 were reached by telephone. Six of the 526 relatives were excluded due to a language barrier or being unaware of the ICU admission. Of the 520 eligible relatives, 329 relatives returned a completed questionnaire (response 63%) (Fig. 1). During the first COVID-19 wave, 72.3% of the relatives were from an ICU where the support was primarily provided by a FST. The majority of relatives were the partner of the patient (52.3%), women (71.6%), 51 years or older (65.3%), medium or highly educated (80.9%), and had a Dutch cultural background (91.5%) (Table 2). In the two COVID-19 groups, 40.1% of relatives suffered from a COVID-19 infection themselves during the ICU admission of the patient. Admitted patients were mostly men (67.8%), 66 years or older (49.2%) and stayed at the ICU for 11 days or longer (62.9%). In total 27.4% of the patients died during their ICU admission. Significant differences between pre-COVID-19, the first COVID-19 wave and the second wave were found for relatives’ and patients’ gender, relative’s cultural background, and whether the patient was transferred to or from another ICU. In addition, non-response analysis showed that relatives who did not fill in the questionnaire were more often the child of the patient (45.3%) and less often the partner (34.1%) compared to relatives who did fill in the questionnaire (respectively 31.9% and 52.3%). There were no statistically significant differences in gender of the patient and whether the patient had deceased or not between relatives who did and who did not participate.

**Table 2:** Relative and patient demographic characteristics (absolute numbers and rounded percentages)

	**Pre-COVID-19 (*n* = 95)**	**First COVID-19 wave (*n* = 130)**	**Second COVID-19 wave (*n* = 104)**	**Total (*n* = 329)**	***p*-value**
*Relative characteristics*					
Bereaved relative	25 (26.3)	39 (30.0)	26 (25.0)	90 (27.4)	0.674
Kinship to patient					0.571
Partner	49 (51.6)	72 (55.4)	51 (49.0)	172 (52.3)	
Child	27 (28.4)	41 (31.5)	37 (35.6)	105 (31.9)	
Other	19 (20.0)	17 (13.1)	16 (15.4)	52 (15.8)	
Gender^a^					**0.047**
Man	36 (37.9)	31 (24.0)	25 (24.3)	92 (28.1)	
Woman	59 (62.1)	98 (76.0)	77 (74.8)	234 (71.6)	
Other	0 (0)	0 (0)	1 (1.0)	1 (0.3)	
Age					0.121
< 30 years	2 (2.1)	5 (3.8)	6 (5.8)	13 (4.0)	
30–50 years	25 (26.3)	38 (29.2)	38 (36.5)	101 (30.7)	
51–65 years	38 (40.0)	56 (43.1)	45 (43.3)	139 (42.2)	
66 years or older	30 (31.6)	31 (23.8)	15 (14.4)	76 (23.1)	
Level of education^a^					0.088
None	2 (2.1)	0 (0)	4 (3.9)	6 (1.9)	
Low	14 (14.9)	26 (20.5)	16 (15.5)	56 (17.3)	
Medium	44 (46.8)	50 (39.4)	54 (52.4)	148 (45.7)	
High	34 (36.2)	51 (40.2)	29 (28.2)	114 (35.2)	
Cultural background^e^					
Dutch	91 (95.8)	124 (95.4)	86 (82.7)	301 (91.5)	** < 0.001**
Other^b^	5 (5.3)	12 (9.2)	22 (21.2)	39 (11.9)	**0.001**
COVID-19 during ICU admission	0^c^	54 (42.2)	39 (37.5)	93 (40.1)^d^	0.535
*Patient characteristics*					
Gender					**0.017**
Man	54 (56.8)	95 (74.8)	72 (69.2)	221 (67.8)	
Woman	41 (43.2)	32 (25.2)	32 (30.8)	105 (32.2)	
Age^a^					0.152
< 30 years	1 (1.1)	1 (0.8)	2 (1.9)	4 (1.2)	
30–50 years	7 (7.4)	12 (9.2)	11 (10.6)	30 (9.1)	
51–65 years	36 (37.9)	52 (40.0)	45 (43.3)	133 (40.4)	
66 years or older	51 (53.7)	65 (50.0)	46 (44.2)	162 (49.2)	
ICU length of stay					0.241
3–5 days	7 (7.4)	5 (3.8)	2 (1.9)	14 (4.3)	
5–10 days	34 (35.8)	36 (27.7)	38 (36.5)	108 (32.8)	
11–20 days	25 (26.3)	46 (35.4)	37 (35.6)	108 (32.8)	
> 20 days	29 (30.5)	43 (33.1)	27 (26.0)	99 (30.1)	
Transferred to/from another ICU	16 (17.0)	59 (45.1)	29 (27.9)	104 (31.7)	** < 0.001**

Missing values: gender relative 2, education 5, COVID-19 2, gender patient 3, transfer 1

^a^Fisher’s exact test instead of chi-squared test, because > 20% of the cells had expected count of less than 5

^b^e.g. Surinamese and Moroccan

^c^value assumed because of pre-COVID-19

^d^total calculated based on groups with valid data on this variable

^e^multiple answers possible

### Elements of support

The elements that the support consisted of are described below including significant differences between the three time periods. The elements are categorised into who, when, how, what and visitation policies.

#### Who provided the support?

During the first COVID-19 wave, the number of relatives receiving support from attending ICU nurses (87.7%) and attending ICU physicians (65.4%) was significantly different from pre-COVID-19 (respectively 97.7% and 90.5%) and the second wave (respectively 96.2% and 83.7%) (Table 3). Also non-ICU healthcare professionals, such as FST members, provided support to a considerable number of relatives from the first wave (54.6%), which was significantly more often compared to the second COVID-19 wave (8.7%). Furthermore, 23.8% of relatives from the first wave received support from a psychosocial caregiver such as a spiritual caregiver, medical social worker or psychologist. This did not differ significantly between the time periods.

**Table 3:** Elements of support per time period (absolute numbers and rounded percentages)

	**Pre-COVID-19 (*n* = 95)**	**First COVID-19 wave (*n* = 130)**	**Second COVID-19 wave (*n* = 104)**	**Total (*n* = 329)**	***p*-value**
**WHO**					
Received support from a support team	0^b^	94 (72.3)	0^c^	94 (28.6)	** < 0.001**
Support received from..^g^					
Attending ICU nurse	93 (97.9)	114 (87.7)	100 (96.2)	307 (93.3)	**0.004**
Attending ICU physician	86 (90.5)	85 (65.4)	87 (83.7)	258 (78.4)	** < 0.001**
Non-ICU healthcare professional	N/A	71 (54.6)	9 (8.7)	80 (34.2)	** < 0.001**
Psychosocial caregiver	29 (30.5)	31 (23.8)	25 (24.0)	85 (25.8)	0.477
Other^a^	1 (1.1)	0 (0)	0 (0)	1 (0.3)	0.289
**WHEN**					
Frequency of contact					**0.050**
Less than 1 time per day	7 (7.4)	3 (2.3)	5 (4.8)	15 (4.6)	
1 time per day	32 (34.0)	55 (42.3)	33 (31.7)	120 (36.6)	
> 1 time per day	41 (43.6)	66 (50.8)	53 (51.0)	160 (48.8)	
Other	14 (14.9)	6 (4.6)	13 (12.5)	33 (10.1)	
Fixed timeslot for contact	5 (5.3)	80 (62.0)	19 (18.3)	104 (31.7)	** < 0.001**
**HOW**					
Method of contact^g^					
Healthcare professional called relative	N/A	99 (78.6)	34 (32.7)	133 (57.5)^d^	** < 0.001**
Relative called healthcare professional	N/A	64 (50.8)	68 (65.4)	132 (57.4)^d^	**0.032**
Other^a^	N/A	3 (2.4)	2 (1.9)	5 (2.2)^d^	1.000
**WHAT**					
Relative received information (e.g. leaflets) about..^g^					
Working method ICU	73 (80.2)	89 (70.1)	69 (67.0)	231 (72.0)	0.106
Visitation policy	90 (96.8)	89 (73.6)	96 (92.3)	275 (86.5)	** < 0.001**
Isolation measures	N/A	79 (62.7)	82 (79.6)	161 (70.3)	**0.006**
Patient’s disease	88 (95.7)	67 (52.3)	53 (52.0)	208 (64.6)	** < 0.001**
Available support	42 (47.7)	65 (51.2)	34 (33.3)	141 (44.3)	**0.017**
Topics discussed in conversations with healthcare professionals^g^					
General COVID-19 information	0^b^	26 (20.2)	17 (16.5)	43 (18.5)^d^	0.501
General ICU information	26 (27.4)	31 (24.0)	26 (25.2)	83 (25.4)	0.863
Changes in medical situation	94 (98.9)	125 (96.9)	101 (98.1)	320 (97.9)	0.642
Physical examination and results	74 (77.9)	101 (78.3)	70 (68.0)	245 (74.9)	0.146
Treatment	82 (86.3)	112 (86.8)	82 (79.6)	276 (84.4)	0.280
Complications	52 (54.7)	93 (72.1)	51 (49.5)	196 (59.9)	**0.001**
Stopping treatment	37 (38.9)	42 (32.6)	32 (31.1)	111 (33.9)	0.478
PICS(-F)	10 (10.5)	11 (8.5)	5 (4.9)	26 (8.0)	0.332
Worries about the patient	47 (49.5)	76 (58.9)	51 (49.5)	174 (53.2)	0.251
Worries about own wellbeing and of relatives and how to inform them	11 (11.6)	52 (40.3)	15 (14.6)	78 (23.9)	** < 0.001**
Other^a^	0 (0)	5 (3.9)	1 (1.0)	6 (1.8)	0.113
Video calling between patient and relative possible	30 (32.3)	110 (86.6)	71 (71.0)	211 (65.9)	** < 0.001**
Being offered emotional support	60 (63.2)	91 (74.0)	70 (68.8)	221 (69.1)	0.239
**VISITIATION POLICY**					
Visitors allowed	95 (100)^b^	15 (11.5)	103 (99.0)	213 (64.7)	** < 0.001**
Allowed to say goodbye in person^a,e^	25 (100)^b^	37 (94.9)	24 (96.0)	86 (96.6)	0.785
Saying goodbye in a private room^f^	17 (89.5)	19 (54.3)	17 (73.9)	53 (68.8)	**0.024**

Missing values: frequency of contact 1, fixed timeslot 1, method of contact 4, working method ICU 8, visitation policy 11, isolation measures 5, patient’s disease 7, available support 11, all topics 2, video calling 9, emotional support 9, allowed to say goodbye 1, saying goodbye private room 1

*N/A* Not asked

^a^Fisher’s exact test instead of chi-squared test, because > 20% of the cells had expected count of less than 5

^b^value assumed based on knowledge on how support was organised pre-COVID-19

^c^value assumed based on knowledge on how support was organised during the second COVID-19 wave

^d^total calculated based on groups with valid data on this variable

^e^only asked to bereaved relatives (*n* = 90)

^f^only asked to bereaved relatives who said goodbye in person (*n* = 78)

^g^multiple answers possible

#### When did relatives receive support?

During the first COVID-19 wave, most relatives had contact with healthcare professionals once a day (42.3%) or more than once a day (50.8%). The proportion of relatives that received support ≥ 1 time per day was significantly higher during the first wave, compared to pre-COVID-19 and the second wave (93.1% versus 77.6% and 82.7%). During the first wave, 62.0% of the relatives received this support at a fixed timeslot, e.g. between 2 and 4 pm. This was significantly different from pre-COVID-19 (5.3%) and the second wave (18.3%).

#### How was the support organised?

During the first COVID-19 wave all contact between relatives and healthcare professionals had to be done via telephone, due to the visitation restrictions. Contact by phone was also a primary method of contact during the second COVID-19 wave. A total of 78.6% of relatives was called by a healthcare professional during the first wave, and 50.8% of relatives called the healthcare professional themselves. Both differed significantly from the second COVID-19 wave (respectively 32.7% and 65.4%).

#### What support did relatives receive?

Topics that relatives most frequently received written or oral information about during the first COVID-19 wave were the visitation policy (73.6%), followed by the working method in the ICU (70.1%), the isolation measures in the ICU (62.7%), background information about the patient’s disease (52.3%) and the support that was available for relatives (51.2%). There were significant differences between the time periods in the proportion of relatives that received information on these topics. Furthermore, in conversations with healthcare professionals relatives during the first wave mostly discussed topics related to the medical status of the patient, such as changes in the medical situation of the patient (96.9%), the treatment provided (86.8%), and performed examinations with corresponding results (e.g. lab results and scans) (78.3%). Least discussed in these conversations were Post Intensive Care Syndrome (PICS) and PICS-Family (PICS-F) (8.5%), and general COVID-19 and ICU information (respectively 20.2% and 24.0%). Discussion of complications that a patient suffered from was the only topic that differed significantly between the first COVID-19 wave (72.1%), and pre-COVID-19 (54.7%) and the second wave (49.5%). During the first wave, the majority of relatives could video call with the patient when the relative could not be at the ICU (86.6%), which differed significantly from pre-COVID-19 (32.3%) and the second wave (71.0%). Finally, during the first wave 74.0% of the relatives were offered emotional support during the ICU admission, which did not differ significantly from the other two time periods.

#### Visitation policies

During the first COVID-19 wave few relatives were allowed to visit the patient at the ICU (11.5%), which differed significantly from pre-COVID-19 (100.0%) and the second wave (99.0%). However, in end-of-life cases nearly all relatives were allowed to say goodbye in person during the first wave (94.9%), with no difference compared to the other two time periods. Of these relatives who said goodbye in person during the first wave 54.3% could do this in a private room without other patients, which differed significantly from the other two time periods (89.5% and 72.9%).

### Experiences and satisfaction with support

Most relatives were positive about the support they received during the ICU admission (Table 4). A total of 82.3% of the relatives from the first COVID-19 wave was satisfied with the frequency of information and 78.0% of the relatives with the timing of information. Results from the questions from the CQI R-ICU show that 96.1% of the relatives from the first wave reported that they received comprehensible information, 96.0% received no contradictory information, 98.4% felt being taken seriously by the healthcare professional, 92.1% reported that healthcare professionals had enough time for them, and 96.1% reported that healthcare professionals listened carefully. Attending ICU nurses, attending ICU physicians, non-ICU healthcare professionals and psychosocial caregivers were all rated with a median score of 9.0 (IQR 2.0) by relatives from the first wave. They rated the support around end-of-life care with a median score of 8.0 (IQR 2.0). No statistically significant differences between the three time periods were found for nearly all experience and satisfaction outcomes. However, significantly more relatives from the first wave felt that healthcare professionals had enough time for them compared to pre-COVID-19 and the second wave (92.1% versus 80.6% and 79.8%). Additional analyses demonstrated that there was no significant difference in experiences and satisfaction between those relatives from the first wave who were primarily support by a FST and those who were supported by ICU healthcare professionals (Table 5). Also experiences and satisfaction did not differ significantly between relatives of whom the patient’s admission was shorter or longer ago (5–6 months, 7–12 months, > 12 months) (Additional file 2).

**Table 4:** Experiences and satisfaction of relatives with support per time period (absolute numbers and rounded percentages)

	**Pre-COVID-19 (*n* = 95)**	**First COVID-19 wave (*n* = 130)**	**Second COVID-19 wave (*n* = 104)**	**Total (*n* = 329)**	***p*-value**
Satisfied with frequency of information, n (%)	75 (78.9)	107 (82.3)	85 (81.7)	267 (81.2)	0.820
Satisfied with timing of information, n (%)	75 (81.5)	99 (78.0)	77 (75.5)	251 (78.2)	0.605
Comprehensible information, n (%)	85 (90.4)	124 (96.1)	98 (94.2)	307 (93.9)	0.222
No contradictory information, n (%)^a^	83 (96.5)	119 (96.0)	87 (91.6)	289 (94.8)	0.267
Felt taken seriously, n (%)^a^	87 (94.6)	125 (98.4)	98 (95.1)	310 (96.3)	0.240
Enough time, n (%)	75 (80.6)	117 (92.1)	83 (79.8)	275 (84.9)	**0.013**
Listened carefully, n(%)	82 (89.1)	123 (96.1)	93 (89.4)	298 (92.0)	0.080
**Scores for multiple types of healthcare professionals and for support around end-of-life care (range 1–10), median (IQR)**^**b**^					
Attending ICU nurse (*n* = 302)	8.0 (1.0)	9.0 (2.0)	9.0 (1.0)	9.0 (2.0)	0.052
Attending ICU physician score (*n* = 249)	8.0 (1.0)	9.0 (2.0)	8.0 (1.0)	8.0 (2.0)	0.224
Non-ICU healthcare professional (*n* = 65)	N/A	9.0 (2.0)	9.0 (1.0)	9.0 (2.0)	0.777
Psychosocial caregivers score (*n* = 85)	7.5 (2.0)	9.0 (2.0)	8.0 (2.0)	9.0 (2.0)	0.091
Support around end-of-life care score (*n* = 90)^c^	8.0 (4.0)	8.0 (2.0)	8.0 (2.0)	8.0 (2.0)	0.726

Missing values or answered not applicable: satisfied with timing of information 8, comprehensible information 2, contradictory information 24, felt taken serious 7, enough time 5, listened carefully 5, attending ICU nurse 5, attending ICU physician 9, non-ICU healthcare professional 15, psychosocial caregivers 26, support around end-of-life care 10

^a^Fisher’s exact test instead of chi-squared test, because > 20% of the cells had expected count of less than 5

^b^scores only included for relatives who said to have received support from this type of healthcare professional, numbers are included behind the variable description

^c^only asked to bereaved relatives

**Table 5:** Experiences and satisfaction of relatives with support for relatives from the first COVID-19 wave who were or were not supported by a support team (absolute numbers and rounded percentages)

	**No support team (*n* = 36)**	**Support team (*n* = 94)**	**Total (*n* = 130)**	***p*-value**
Satisfied with frequency of information, n (%)	30 (83.3)	77 (81.9)	107 (82.3)	1.000
Satisfied with timing of information, n (%)	29 (82.9)	70 (76.1)	99 (78.0)	0.480
Comprehensible information, n (%)^a^	34 (97.1)	90 (95.7)	124 (96.1)	1.000
No contradictory information, n (%)^a^	32 (94.1)	87 (96.7)	119 (96.0)	0.614
Felt taken seriously, n (%)^a^	34 (100.0)	91 (97.8)	125 (98.4)	1.000
Enough time, n (%)^a^	33 (97.1)	84 (90.3)	117 (92.1)	0.287
Listened carefully, n (%)^a^	34 (100)	89 (94.7)	123 (96.1)	0.324
**Scores for multiple types of healthcare professionals and for support around end-of-life care (range 1–10), median (IQR)**^**b**^				
Attending ICU nurse score (*n* = 112)	9.0 (2.0)	9.0 (2.0)	9.0 (2.0)	0.955
Attending ICU physician score (*n* = 80)	9.0 (2.0)	9.0 (3.0)	9.0 (2.0)	0.211
Psychosocial caregivers score (*n* = 24)	8.5 (3.0)	9.0 (2.0)	9.0 (2.0)	0.526
Support around end-of-life care score (*n* = 37)^c^	8.0 (1.0)	8.0 (2.0)	8.0 (2.0)	0.595

Missing values: satisfied with timing of information 3, comprehensible information 1, contradictory information 6, felt taken serious and enough time 3, listened carefully 2, attending ICU nurse 2, attending ICU physician 5, psychosocial caregivers 7, support around end-of-life care 2

^a^Fisher’s exact test instead of chi-squared test, because > 20% of the cells had expected count of less than 5

^b^scores only included for relatives who said to have received support from this type of healthcare professional, numbers are included behind the variable description

^c^only asked to bereaved relatives

### Associations between elements of support and experiences and satisfaction with the support

Elements of support were tested for associations with several of the previously mentioned experience and satisfaction outcomes (Table 6). Seven elements were significantly associated with one or more of the experience and satisfaction outcomes: 1) receiving support from an attending ICU physician, 2) fixed timeslot, 3) the healthcare professionals taking the initiative to call the relative, 4) the number of topics that relatives received information on, 5) the number of topics discussed in conversations with healthcare professionals, 6) video calling between a patient and relative(s), and 7) being offered emotional support. Element 4, 5 and 7 were statistically significant associated with the highest number of experience and satisfaction outcomes, namely minimally four. These three elements were all associated with satisfaction with the frequency of information, satisfaction with the timing of information, healthcare professionals having enough time and healthcare professionals listening carefully. In addition, element 4 and 7 were also associated with feeling being taken seriously. Finally, element four was associated with receiving comprehensible information. Six analysed elements of support were not significantly associated with any of the outcome measures, namely receiving support from an attending ICU nurse, receiving support from a support team, receiving support from a psychosocial caregiver, the frequency of contact, the relative taking the initiative to call the healthcare professional or another method of contact between the healthcare professional and relative.

**Table 6:** Associations between elements of support and experiences and satisfaction with the support, adjusted for age and gender of the respondent

			**Satisfied with frequency of information (*n* = 329)**			**Satisfied with timing of information (*n* = 321)**				
			**Row %**		**OR (95% CI)**	**Row %**	**OR (95% CI)**			
**WHO**										
Support received from attending ICU nurse										
No (*n* = 22)			72.7		1.00	81.8	1.00			
Yes (*n* = 307)			81.8		1.84 (0.68–4.99)	77.9	0.75 (0.24–2.30)			
Support received from attending ICU physician										
No (*n* = 71)			69.0		1.00	71.8	1.00			
Yes (*n* = 258)			84.5		**2.58 (1.40–4.77)**	80.0	1.60 (0.87–2.95)			
Support received from non-ICU healthcare professional^a^										
No (*n* = 154)			79.9		1.00	75.3	1.00			
Yes (*n* = 80)			86.3		1.58 (0.74–3.39)	79.7	1.31 (0.67–2.56)			
Support received from psychosocial caregiver										
No (*n* = 245)			79.5		1.00	76.5	1.00			
Yes (*n* = 84)			85.9		1.49 (0.75–2.98)	83.1	1.46 (0.76–2.80)			
**WHEN**										
Frequency of contact										
Less than 1 time per day (*n* = 15)			66.7		1.00	86.7	1.00			
1 time per day (*n* = 120)			77.5		1.66 (0.51–5.37)	78.6	0.59 (0.12–2.82)			
> 1 time per day (*n* = 160)			90.6		4.89 (1.45–16.51)^c^	80.0	0.69 (0.15–3.24)			
Other (*n* = 33)			57.6		0.71 (0.19–2.58)	59.4	0.24 (0.05–1.26)			
Fixed timeslot for contact										
No (*n* = 224)			75.9		1.00	72.4	1.00			
Yes (*n* = 104)			92.3		**3.72 (1.69–8.17)**	90.3	**3.70 (1.80–7.64)**			
**HOW**										
Healthcare professional called relative^a^										
No (*n* = 97)			74.2		1.00	67.4	1.00			
Yes (*n* = 133)			87.2		2.11 (1.05–4.24)^c^	83.1	2.24 (1.18–4.24)^c^			
Relative called healthcare professional^a^										
No (*n* = 98)			84.7		1.00	79.4	1.00			
Yes (*n* = 132)			79.5		0.76 (0.37–1.54)	74.2	0.76 (0.40–1.44)			
Contact between healthcare professional and relative different method^a^										
No (*n* = 225)			81.8		1.00	76.4	1.00			
Yes (*n* = 5)			80.0		1.12 (0.12–10.40)	80.0	1.47 (0.16–13.58)			
**WHAT**										
Number of topics relative received information about (e.g. leaflets)										
≤ 1 (*n* = 54)			59.3		1.00	55.6	1.00			
2–3 (*n* = 126)			79.4		**2.92 (1.43–5.97)**	80.0	**3.32 (1.62–6.78)**			
4–5 (*n* = 149)			90.6		**7.42 (3.33–16.54)**	85.0	**4.67 (2.26–9.64)**			
Number of topics discussed with healthcare professionals										
1–3 (*n* = 92)			66.3		1.00	67.0	1.00			
4–5 (*n* = 122)			79.5		2.18 (1.16–4.10)^c^	76.5	1.65 (0.88–3.08)			
6–9 (*n* = 113)			95.6		**13.19 (4.79–36.36)**	89.3	**4.38 (2.05–9.37)**			
Video calling between patient and relative possible										
No (*n* = 109)			72.5		1.00	75.2	1.00			
Yes (*n* = 211)			85.3		**2.56 (1.42–4.60)**	79.2	1.32 (0.75–2.34)			
Being offered emotional support										
No (*n* = 99)			66.7		1.00	65.3	1.00			
Yes (*n* = 221)			87.3		**3.39 (1.89–6.09)**	83.4	**2.55 (1.46–4.46)**			
	**Comprehensible information (*n* = 327)**		**No contradictory information (*n* = 305)**		**Felt taken seriously (*n* = 322)**		**Enough time (*n* = 324)**		**Listening carefully (*n* = 324)**	
	**Row %**	**OR (95% CI)**	**Row %**	**OR (95% CI)**	**Row %**	**OR (95% CI)**	**Row %**	**OR (95% CI)**	**Row %**	**OR (95% CI)**
**WHO**										
Support received from attending ICU nurse										
No (*n* = 22)	95.2	1.00	90.0	1.00	90.5	1.00	95.2	1.00	95.2	1.00
Yes (*n* = 307)	93.8	0.69 (0.09–5.47)	95.1	2.95 (0.59–14.74)	96.7	2.90 (0.57–14.67)	84.2	0.30 (0.04–2.32)	91.7	0.56 (0.07–4.38)
Support received from attending ICU physician										
No (*n* = 71)	90.1	1.00	89.4	1.00	91.3	1.00	82.9	1.00	87.0	1.00
Yes (*n* = 258)	94.9	2.07 (0.79–5.45)	96.2	3.30 (1.15–9.44)^c^	97.6	4.38 (1.34–14.37)^c^	85.4	1.31 (0.63–2.69)	93.3	2.25 (0.94–5.36)
Support received from non-ICU healthcare professional^a^										
No (*n* = 154)	93.5	1.00	91.5	1.00	96.7	1.00	81.6	1.00	91.4	1.00
Yes (*n* = 80)	98.8	5.52 (0.69–44.41)	98.7	7.80 (0.96–63.71)	97.5	1.43 (0.27–7.78)	96.2	6.20 (1.76–21.81)^c^	96.3	2.14 (0.58–7.89)
Support received from psychosocial caregiver										
No (*n* = 245)	93.4	1.00	95.6	1.00	95.4	1.00	83.0	1.00	90.9	1.00
Yes (*n* = 84)	95.2	1.28 (0.41–3.99)	92.3	0.51 (0.18–1.48)	98.8	3.78 (0.48–30.05)	90.4	1.82 (0.81–4.09)	95.1	1.85 (0.61–5.59)
**WHEN**										
Frequency of contact										
Less than 1 time per day (*n* = 15)	92.9	1.00	83.3	1.00	84.6	1.00	69.2	1.00	85.7	1.00
1 time per day (*n* = 120)	92.4	1.24 (0.14–10.99	93.5	2.45 (0.42–14.22)	98.3	15.15 (1.72–133.72)^c^	84.0	2.27 (0.62–8.35)	91.6	2.19 (0.41–11.65)
> 1 time per day (*n* = 160)	97.5	3.77 (0.38–37.53)	97.4	6.48 (1.00–41.97)^c^	98.8	23.48 (2.67–206.62)^c^	91.8	4.83 (1.28–18.32)^c^	96.9	5.30 (0.90–31.12)
Other (*n* = 33)	81.8	0.42 (0.04–3.96)	90.3	1.60 (0.22–11.74)	80.6	1.06 (0.16–6.90)	62.5	0.73 (0.18–2.95)	71.0	0.42 (0.08–2.31)
Fixed timeslot for contact										
No (*n* = 224)	91.4	1.00	94.2	1.00	94.9	1.00	81.7	1.00	90.0	1.00
Yes (*n* = 104)	99.0	10.90 (1.42–83.43)^c^	95.9	1.32 (0.41–4.26)	99.0	5.78 (0.73–45.79)	91.3	2.18 (1.01–4.72)^c^	96.1	2.65 (0.88–7.99)
**HOW**										
Healthcare professional called relative^a^										
No (*n* = 97)	90.7	1.00	92.5	1.00	93.7	1.00	76.0	1.00	88.5	1.00
Yes (*n* = 133)	98.5	6.52 (1.34–31.78)^c^	95.1	1.54 (0.48–4.97)	99.2	7.12 (0.82–61.62)	93.9	**6.12 (2.38–15.73)**	96.2	4.07 (1.23–13.48)^c^
Relative called healthcare professional^a^										
No (*n* = 98)	94.8	1.00	93.5	1.00	95.9	1.00	88.7	1.00	92.8	1.00
Yes (*n* = 132)	95.5	1.13 (0.32–3.96)	94.3	1.32 (0.42–4.20)	97.7	2.10 (0.45–9.94)	84.6	0.69 (0.30–1.56)	93.9	1.23 (0.42–3.58)
Contact between healthcare professional and relative different method^a^										
No (*n* = 225)	95.1		94.3		96.8		86.0		92.8	
Yes (*n* = 5)	100	b	80.0	0.24 (0.02–2.49)	100	b	100	b	100	b
**WHAT**										
Number of topics relative received information about (e.g. leaflets)										
≤ 1 (*n* = 54)	83.3	1.00	93.6	1.00	84.9	1.00	71.7	1.00	78.8	1.00
2–3 (*n* = 126)	94.4	3.09 (1.07–8.94)^c^	92.4	0.96 (0.24–3.82)	97.5	7.96 (1.94–32.65)^c^	83.1	2.46 (1.11–5.43)^c^	91.3	3.13 (1.22–8.02)^c^
4–5 (*n* = 149)	97.3	**6.28 (1.81–21.80)**	97.1	2.79 (0.57–13.54)	99.3	**28.04 (3.32–236.69)**	91.2	**5.16 (2.17–12.29)**	97.3	**9.76 (2.89–32.95)**
Number of topics discussed with healthcare professionals										
1–3 (*n* = 92)	89.0	1.00	92.5	1.00	91.0	1.00	71.1	1.00	83.0	1.00
4–5 (*n* = 122)	95.1	2.35 (0.81–6.78)	95.7	1.90 (0.55–6.55)	98.3	6.20 (1.26–30.42)	87.6	**3.09 (1.49–6.44)**	95.1	3.92 (1.42–10.76)^c^
6–9 (*n* = 113)	96.5	3.24 (0.96–10.90)	95.3	1.80 (0.52–6.29)	98.2	6.14 (1.24–30.45)	93.8	**7.03 (2.81–17.61)**	96.5	**5.79 (1.80–18.63)**
Video calling between patient and relative possible										
No (*n* = 109)	91.6	1.00	95.9	1.00	95.2	1.00	78.1	1.00	87.6	1.00
Yes (*n* = 211)	95.3	1.93 (0.74–5.02)	94.0	0.73 (0.23–2.37)	96.7	1.73 (0.52–5.79)	88.1	2.24 (1.17–4.31)^c^	93.8	1.99 (0.86–4.62)
Being offered emotional support										
No (*n* = 99)	87.8	1.00	92.5	1.00	88.3	1.00	70.5	1.00	82.3	1.00
Yes (*n* = 221)	96.4	3.66 (1.43–9.40)^c^	95.6	1.93 (0.68–5.51)	99.5	**26.10 (3.30–206.32)**	90.5	**4.46 (2.30–8.63)**	95.9	**5.71 (2.35–13.90)**

Missing values (ranges): frequency of contact 1, fixed timeslot 1, healthcare professional called relative 4, relative called healthcare professional 4, other method of contact 4, number of topics discussed with healthcare professionals 1–2, video calling 8–9, emotional support 6–9

Bold values indicate odds ratios that are statistically significant using Bonferroni adjusted alpha levels of .0038

^a^only asked to relatives from the first and second COVID-19 wave (*n* = 234)

^b^not included in analyses because all relatives who had a different method of contact were satisfied and none were not satisfied

^c^Odds ratios in which 1 is not included in the 95% confidence interval, but no statistically significant association using Bonferroni adjusted alpha levels of .0038

## Discussion

This quantitative multicentre cohort study offers new insights into which elements of support are associated with positive experiences with the support in the ICU. As expected, the way in which the support for relatives of ICU patients was organised during the first COVID-19 wave differed significantly from pre-COVID-19 and the second wave. Differences were found in all categories of elements of support: who, when, how and what. For instance, relatives from the first wave reported higher frequencies of contact and having a fixed timeslot for contact was much more common for these relatives. However, these differences in the organisation of support do not seem to translate into differences in experiences and satisfaction with the received support. Overall, relatives from the three time periods were very positive about the support. The only difference in experience and satisfaction between the three time periods, was the proportion of relatives indicating that healthcare professionals had enough time for them, which was significantly higher among relatives from the first wave compared to the other two time periods. Individual elements of support which were associated with many positive experiences and satisfaction across the three time periods include: fixed timeslot, receiving information (e.g. leaflets) on ≥ 2 topics, discussing > 5 topics with healthcare professionals, and being offered emotional support.

### The alternative support during the COVID-19 pandemic is highly appreciated

As previously mentioned, relatives in our study were very positive about the support they received. These findings align with other studies from before and during the pandemic reporting high satisfaction with ICU care and also specifically with communication [20–22]. For example, ninety-nine percent of relatives in a French ICU were satisfied with the manner and frequency with which they received information during the COVID-19 pandemic [23]. Healthcare professionals and researchers were concerned that the quality of support for relatives of ICU patients would be compromised during the COVID-19 pandemic [11, 24, 25], but this is not reflected in our results. Although relatives were not allowed to visit their loved one and all communication was via telephone or video calling during the first wave, more than 75% of relatives showed high scores on our experience and satisfaction outcomes. These numbers did not differ significantly from relatives from pre-COVID-19 and the second wave. Strikingly, relatives from the first wave were even more positive about healthcare professionals having enough time for them compared to the other two time periods. Considering the volume of patients and the high workload for healthcare professionals in the first months of the pandemic, this might be contradictory to what was expected. We would like to discuss two possible explanations for this finding. Firstly, due to the visitation restrictions most ICU healthcare professionals were probably more aware of the importance of adequate support for relatives. As a result, the ICU healthcare professionals explicitly dedicated time to this task despite the high workload or arranged FSTs dedicated to this task. A study which evaluated a FST in the ICU in the United Kingdom also found that relatives were mostly very or extremely satisfied [14]. The absence of time pressure when speaking with a FST member was also highly appreciated by relatives as reported in a qualitative study by Klop et al. [13]. The second explanation is related to expectations that relatives may have had during the first wave. Relatives were probably well aware of the crisis situation in the ICU, and given the situation they were satisfied with the provided support. The latter explanation is in line with a qualitative study that reported an awareness among relatives of the situation and an appreciation for time that healthcare professionals nevertheless took for regular updates [21].

### Elements of support associated with positive experiences

Multiple elements of support were found to be associated with positive experiences and satisfaction, for example daily or more frequent contact initiated by healthcare professionals. Some elements have also been discussed in other studies, such as emotional support. Emotional support seems to be an important element of support for relatives in the ICU as we found that being offered emotional support is associated with many of the positive experience and satisfaction measures, which is in accordance with findings from Stricker et al. [7]. However, we also found that not all relatives were actually offered this support, possibly due to healthcare professionals underestimating the need for emotional support for relatives [26]. All of this suggests that more attention and further research is needed into the topic of emotional support for relatives of ICU patients. Furthermore, several qualitative studies reported that relatives’ satisfaction was related to the predictability and certainty of calls at a fixed moment [11, 13, 21]. Similarly, we found that a fixed timeslot was significantly associated with being satisfied with the frequency and timing of information. During the first COVID-19 wave a fixed timeslot was relatively common as reported by 62% of the relatives, but pre-COVID-19 only 5% of relatives had a fixed timeslot and during the second wave 18%. This number from the first wave is slightly higher in Dutch ICUs compared to Scandinavian ICUs, where 44% had a set time for the conversations [17]. Waiting for an update about your loved one in the ICU, and not knowing when to expect that update can be very stressful [18]. Therefore, some researchers have urged consideration of using fixed timeslots in future crisis situations [13, 18]. Considering the positive experiences associated with using a fixed timeslot, applying this in “normal” daily practice could also be valuable.

### Strengths and limitations

The current study has several strengths and limitations that need to be acknowledged. First of all, our study is one of the first studies on support for relatives in the ICU during COVID-19 to include three time periods, as we also included the pre-COVID-19 period and the second COVID-19 wave. In addition, we included a great number of elements of support (who, when, how and what) which provides a comprehensive overview of the organisation of support for relatives in the ICU. Yet, this also increases the risk of false-positive associations (Type 1 error). To minimize this risk we used a more conservative alpha level by applying the Bonferroni correction. Important to note is that the period between ICU admission and study participation varied between relatives from the three time periods, with longer periods between admission and participation for the pre-COVID-19 group. Therefore, there might be different degrees of recall bias between the groups. However, we found no significant differences in experiences between relatives of whom the patients ICU admission was shorter or longer ago. Another bias that might be present is non-respondent bias. Analysis showed no difference between responders and non-responders with regards to the gender of the patient and whether the patient had deceased or not, but the kinship to the patient was significantly different. Furthermore, relatives who did not want to participate in the study might be less satisfied about the care their loved one received in the ICU or the support they received themselves. Therefore the positive experiences with support in the current study might be an overestimation. Additionally, we found that relatives from the three time periods differed significantly in several demographic variables, such as relatives’ and patient’s gender and relatives’ cultural background. These differences are likely a result of higher number of males requiring ICU admissions due to a COVID-19 infection compared to females, as well as a higher number of ICU admissions among non-Dutch ethnic groups. The sample size was too small to correct for these variables. The limited sample size also implied that there were small number of relatives with certain characteristics and therefore the resulting confidence intervals are wide. These results should therefore be interpreted with caution.

## Conclusion

Our study provides new insights into how to best organise support for relatives in the ICU. Interestingly, even though the COVID-19 pandemic forced ICUs to considerably change their methods of supporting relatives, the relatives still valued this support as positive as during non-COVID times. As these alternative ways of support were positively experienced, multiple avenues for improvement can be highlighted from our study for future comparable situations as well as for normal daily ICU practice. We recommend daily contact at a fixed timeslot, providing information on and discussing multiple topics (e.g. medical situation as well as relative’s well-being), and offering emotional support. ICUs are strongly encouraged to review their method of support for relatives and to consider which elements of support need improvement in their practice.

## Supplementary Information

**Additional file 1. **Questionnaire (translated).

**Additional file 2.** Differences in experiences and satisfaction for different periods between ICU admission and questionnaire completion.

## Data Availability

The dataset used and/or analysed during the current study is available from the corresponding author on reasonable request.

## Abbreviations

COVID-19: COronaVIrusDisease 2019
CQI R-ICU: Consumer Quality Index Relatives in the ICU
ICU: Intensive Care Unit
FST: Family Support Team
PICS(-F): Post Intensive Care Syndrome(-Family)
UMC: University Medical Center
VSNU: Association of Universities in the Netherlands
SCR: Sophie C. Renckens
HRP: H. Roeline Pasman
H.T. Klop: Hanna T. K
CdP: Chantal du Perron
LvZ: Lia van Zuylen
MAHS: Monique A.H. Steegers
BLtT: Birkitt L. ten Tusscher
FCHA: Floor C.H. Abbink
WdR: Wouter de Ruijter
LCMV: Lilian C.M. Vloet
SCEK: Stephanie C.E. Koster
BDOP: Bregje D. Onwuteaka-Philipsen
