# Supplementary material for: Support for relatives in the intensive care unit: lessons from a cross-sectional multicentre cohort study during the COVID-19 pandemic
Source: BMC Health Serv Res. 2023 Jul 18;23:763. doi: 10.1186/s12913-023-09756-2 (PMC10353201; doi:10.1186/s12913-023-09756-2)
Supplement: Supplementary file 2 — Additional file 2. Differences in experiences and satisfaction for different periods between ICU admission and questionnaire completion. [file 12913_2023_9756_MOESM2_ESM.docx]

| **Table A1.**  Experiences and satisfaction of relatives with support for with different periods between ICU admission and questionnaire completion (absolute numbers and rounded percentages) | | | | | |
| --- | --- | --- | --- | --- | --- |
|  | **5-6 months (n=27)** | **7-12 months (n=146)** | **>12 months (n=156)** | **Total (n=329)** | **p-value** |
| Satisfied with frequency of information, n (%) | 19 (70.4) | 125 (85.6) | 123 (78.8) | 267 (81.2) | 0.115 |
| Satisfied with timing of information, n (%) | 18 (72.0) | 114 (78.1) | 119 (79.3) | 251 (78.2) | 0.734 |
| Comprehensible information, n (%) | 25 (92.6) | 140 (95.9) | 142 (92.2) | 307 (93.9) | 0.451 |
| No contradictory information, n (%) | 22 (91.7) | 127 (93.4) | 140 (96.6) | 289 (94.8) | 0.375 |
| Felt taken seriously, n (%) | 25 (96.2) | 140 (96.6) | 145 (96.0) | 310 (96.3) | 1.000 |
| Enough time, n (%) | 22 (81.5) | 125 (85.6) | 128 (84.8) | 275 (84.9) | 0.895 |
| Listened carefully, n(%) | 23 (85.2) | 136 (93.2) | 139 (92.1) | 298 (92.0) | 0.389 |
| **Scores for multiple types of healthcare professionals and for support around end-of-life care (range 1-10), median (IQR)^a^** | | | | | |
| Attending ICU nurse (n=302) | 9.0 (2.0) | 9.0 (2.0) | 9.0 (2.0) | 9.0 (2.0) | 0.728 |
| Attending ICU physician score (n=249) | 8.0 (2.0) | 8.0 (2.0) | 8.0 (2.0) | 8.0 (2.0) | 0.809 |
| Healthcare professional who is not involved in the patient‘s care score (n=65) | 9.0 (?) | 9.0 (2.0) | 6.5 (3.0) | 9.0 (2.0) | **0.013** |
| Psychosocial caregivers score (n=59) | 10.0 (?) | 8.0 (2.0) | 8.0 (3.0) | 8.0 (2.0) | 0.361 |
| Support around end-of-life care score (n=80)^b^ | 8.5 (2.0) | 8.0 (2.0) | 8.0 (2.0) | 8.0 (2.0) | 0.336 |
| ^a^= scores only included for respondents who said to have received support from this type of healthcare professional, numbers are included behind the variable description  ^b^=only asked to bereaved relatives  Missing values or answered not applicable: satisfied with timing of information 8, comprehensible information 2, contradictory information 24, felt taken serious 7, enough time 5, listened carefully 5, attending ICU nurse 5, attending ICU physician 9, healthcare professional who is not involved in the patient‘s care 15, psychosocial caregivers 26, support around end-of-life care 10 | | | | | |

**Additional file 2. Differences in experiences and satisfaction for different periods between ICU admission and questionnaire completion**
